# Comparative Approach to Performance Estimation of Pulsed Wave Doppler Equipment Based on Kiviat Diagram

**DOI:** 10.3390/s24196491

**Published:** 2024-10-09

**Authors:** Giorgia Fiori, Andrea Scorza, Maurizio Schmid, Silvia Conforto, Salvatore Andrea Sciuto

**Affiliations:** Department of Industrial, Electronic and Mechanical Engineering, University of Roma Tre, 00146 Rome, Italy; andrea.scorza@uniroma3.it (A.S.); maurizio.schmid@uniroma3.it (M.S.); silvia.conforto@uniroma3.it (S.C.); salvatore.sciuto@uniroma3.it (S.A.S.)

**Keywords:** quality assessment, ultrasound diagnostic systems, pulsed wave Doppler, flow phantom, measurement methods, Kiviat diagram

## Abstract

Quality assessment of ultrasound medical systems is a demanding task due to the high number of parameters to quantify their performance: in the present study, a Kiviat diagram-based integrated approach was proposed to effectively combine the contribution of some experimental parameters and quantify the overall performance of pulsed wave Doppler (PWD) systems for clinical applications. Four test parameters were defined and assessed through custom-written measurement methods based on image analysis, implemented in the MATLAB environment, and applied to spectral images of a flow phantom, i.e., average maximum velocity sensitivity (AMVS), velocity measurements accuracy (VeMeA), lowest detectable signal (LDS), and the velocity profile discrepancy index (VPDI). The parameters above were scaled in a standard range to represent the four vertices of a Kiviat plot, whose area was considered the overall quality index of the ultrasound system in PWD mode. Five brand-new ultrasound diagnostic systems, equipped with linear array probes, were tested in two different working conditions using a commercial flow phantom as a reference. The promising results confirm the robustness of AMVS, VeMeA, and LDS parameters while suggesting further investigations on the VPDI.

## 1. Introduction

The first diagnostic applications of ultrasound in medicine can be dated back to 1942, when the Austrian psychiatrist and neurologist Karl Theo Dussik realized a device to investigate the displacement of the cerebral hemispheres [1]. From then onward, new innovative devices and techniques were continuously developed and are emerging and growing quickly even today [2,3,4,5], including the application of integrated sensing and communications, multidimensional spectral super-resolution [6,7], and artificial intelligence [8,9]. Medical ultrasound is one of the most rapidly advancing imaging modalities and to date is deemed to be a first-line imaging technique, pivotal in both clinical diagnosis and interventional treatment [3,4]. Diagnostic ultrasonography is widespread and largely used in a variety of clinical settings by several professionals [10,11,12,13,14] as it is a real-time powerful tool for imaging anatomical districts and providing functional representations of blood flow. Among diagnostic imaging technologies, ultrasound is recognized by the World Health Organization (WHO) as the safest and least expensive [15], and its major benefits include portability, real-time imaging, and ease of use. The most common functional ultrasound applications make use of the Doppler effect to detect the blood flow in vessels and in the heart, and display it in a spectral form or in a 2D color image, i.e., spectral Doppler and 2D color flow imaging, respectively [16,17]. Concerning spectral Doppler techniques, pulsed wave Doppler (PWD) is frequently used by clinicians to assist in diagnosis, as it allows quantification of blood flow velocity over time from adjusted depths in tissues.

Based on the above considerations, quality assessment (QA) of ultrasound imaging equipment is made necessary to prevent and monitor the progressive worsening in performance over time [18,19]. It is well known that ultrasound imaging equipment must undergo acceptance testing and performance evaluations [20,21]. The former provides the performance baseline of the ultrasound system, and it is performed upon purchase, repair, and replacement of system components, and following major software updates and periods of inactivity. On the other hand, performance evaluations involve periodic checks of the ultrasound system to monitor its performance over time.

As highlighted in [19,22,23,24,25,26,27], degradation of image quality and inaccurate measurements could impact clinical diagnosis, increasing the risk of diagnostic errors in routine clinical practice. Many studies in the scientific literature found a high incidence of defective ultrasound probes in clinical use [28,29,30]. Moreover, overestimations of up to 50% were observed in the measurement of maximum blood velocity [31,32].

The importance of quality assessment in medical ultrasound has been widely recognized in the scientific literature [18,19,33], and medical associations, as well as accrediting bodies, have published guidelines and recommendations concerning ultrasound QA over the years [20,34,35,36]. Despite these attempts, QA is not mandatory in this field and an internationally approved quality standard has yet to be developed [18,19,22,23,33,37,38]. Performance evaluation of Doppler systems is still an open issue and this is also due to the wide variety of test parameters [18,21] and the lack of automatic measurement methods. To the authors’ knowledge, software packages and applications found in the scientific literature of the field for B-mode QA are usually based on a black-box approach and suffer from operator-related errors [38,39]. Therefore, among the main challenges in the field is the definition and development of an effective approach that integrates the contributions of these test parameters into a few quantities that can be quickly understood by the technician [38,39,40]. Concerning this last point, Kiviat diagrams (or Kiviat plots) have been recently proposed as a tool to combine effectively the contribution of experimental test parameters for Color Doppler [38], pulsed wave Doppler [40], and B-mode [39] quality assessment. The performance tests were objectively estimated by image analysis-based methods and the plot area turned out to be a promising index of the overall system performance [38,39,40]. Kiviat diagram representation provided a standardized overview of the experimental parameters and facilitated visualization and comparison of ultrasound diagnostic systems (UDSs) produced by different manufacturers. Indeed, it is widely used in various fields, e.g., economics, engineering, computing, health, as a tool for comparing outcome metrics, conveying a large amount of information [41,42,43].

Based on the first results retrieved in [40], the study herein proposed, for the first time, aims to investigate the Kiviat diagram-based integrated approach for the comparison of PWD ultrasound systems intended for use in clinical care. Five brand-new UDSs, each equipped with a linear array probe, were tested in two working conditions. For performance comparison, four test parameters were experimentally assessed through objective measurement methods based on image analysis implemented in the MATLAB environment. The quantitative parameters, derived from definitions often expressed only in a qualitative way in the scientific literature, are the velocity profile discrepancy index [44,45], average maximum velocity sensitivity [46], velocity measurements accuracy [40], and lowest detectable signal [47,48].

The present study is organized as follows: Section 2 includes the description of the experimental setup, the QA parameters and measurement methods implemented, and the data acquisition protocol and the parameter scaling needed to combine and compare the results. Section 3 deals with the measurement uncertainty analysis. Experimental outcomes are presented in Section 4 and are then discussed in Section 5, which also includes future research directions and conclusions.

## 2. Materials and Methods

### 2.1. Experimental Setup

Five high-technology level ultrasound diagnostic systems, equipped with a linear array probe each, were tested. The systems, manufactured by different companies, are intended for general-purpose imaging in clinical care. As in [38,49], transducers worked at two different working conditions: pre-set A, i.e., a clinical pre-set provided by the product specialist to assume the best performance during the test session, and pre-set B, i.e., a raw pre-set in which pre- and post-processing settings were minimized. General PWD settings adopted for the two working conditions are summarized in Table 1.

The experimental setup also included a commercial reference device. The model used is a self-contained Doppler flow phantom [50] consisting of a non-compliant flow circuit embedded in a tissue-mimicking material (TMM). The hydraulic circuit made up of two tube segments of known size and location is filled with a blood-mimicking fluid (BMF) that is pumped into the vessels at a constant or pulsatile flow rate in an adjustable range. In the diagonal vessel, parabolic flow is achieved at all flow rates [51]. Specifications of the reference device are listed in Table 2.

### 2.2. Test Parameters

#### 2.2.1. Velocity Profile Discrepancy Index

The Doppler sample volume (SV) is a sensitive region placed within the B-mode image where the velocity of blood flow is measured. It is adjusted by the operator in length and depth on the area of interest [52,53]. The velocity profile discrepancy index (VPDI) has been introduced in [44,45] to objectively assess any faults in SV length and registration accuracy. In particular, it quantifies the discrepancy between the expected and measured velocity profile along the vessel section when laminar flow is assumed. The parameter is related to both the actual size and registration error of the sample volume.

**Figure 1:**
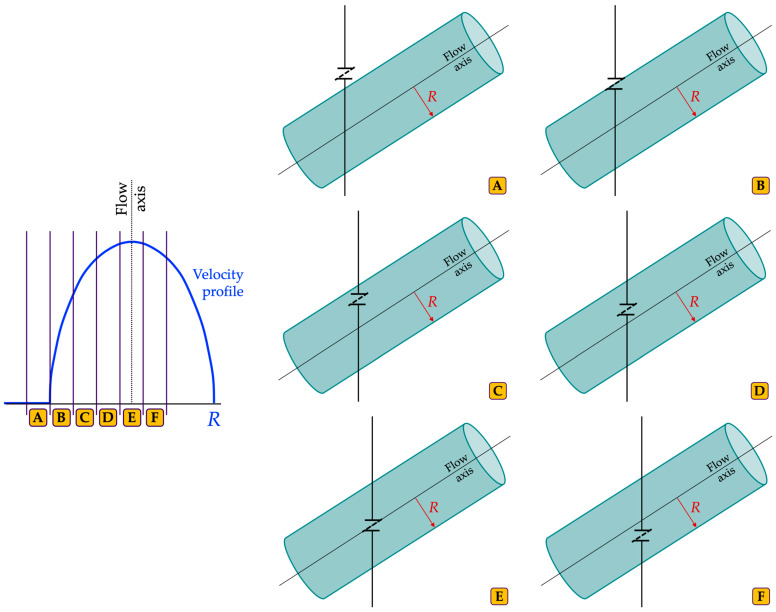
Example of sample volume positioning at six different radial distances (*R* = radius) from the flow axis of a sketched straight vessel of a reference device for the velocity profile measurement.

**Figure 2:**
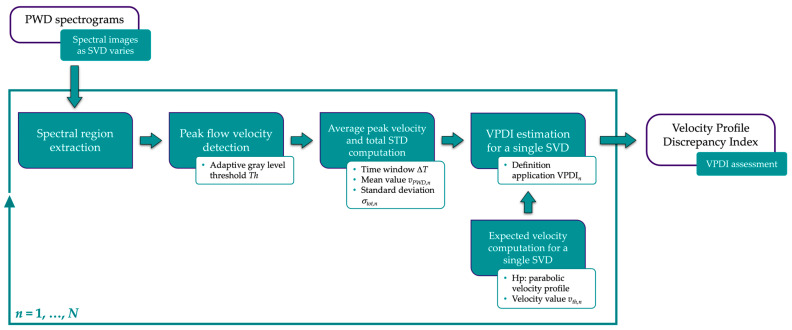
Block diagram of the measurement method for the velocity profile discrepancy index assessment.

The measurement method presented in [44,45] automatically estimates the VPDI by post-processing PWD spectrograms. The spectral images are required to be collected by adjusting the SV at different radial distances from the axis of a straight vessel of a Doppler reference device while keeping the sample volume length (SVL) fixed. As shown in Figure 1, each sample volume depth (SVD) locates a different position with respect to the diameter of the phantom vessel under the hypothesis of a constant velocity profile along its flow axis [44]. The main processing steps of the image analysis-based method are shown in Figure 2 and summarized in the following.

First, the metadata provided in the DICOM (Digital Imaging and Communications in Medicine) standard is used to identify and extract the spectral region from the acquired image. As in [44,45,49], an adaptive threshold *Th* is applied to detect the pixel corresponding to the peak flow velocity for each spectral line. The coordinates of these pixels are then associated with the respective velocity value for each time instant, based on the pre-set full-scale velocity. This second step allows us to objectively derive the maximum flow velocity trend as a function of time. At this point, both the average maximum velocity *v_PWD_* and the standard deviation *σ_PWD_* are computed over a fixed time window Δ*T* corresponding to *L* spectral lines. By repeating the previous steps for *N* spectral images, one for each position within the vessel, the discrepancy index is calculated by applying the mathematical formulation in [44,45]:

(1) $$VPDI=\sum_{n=1}^{N}{VPDI}_{n}=\sum_{n=1}^{N}\frac{{\left(v_{PWD,n}-v_{th,n}\right)}^{2}}{\sigma_{tot,n}^{2}}$$

where *v_th_*_,*n*_ is the velocity derived from the expected parabolic profile for the *n*-th depth of the sample volume identifying a given position with respect to the vessel radius. As reported in [44,45], the computation of *v_th_*_,*n*_ is also dependent on the SVL setting. On the other hand, *σ_tot_*_,*n*_ is the total standard deviation (STD) estimated for the *n*-th SVD by combining the following uncertainty contributions [44,45]:

(2) $$\left\{\begin{array}{c} \sigma_{tot,n}=\sqrt{\sigma_{PWD,n}^{2}+\sigma_{rand,n}^{2}+\sigma_{th,n}^{2}}\;\mathrm{inside}\;\mathrm{the}\;\mathrm{vessel}\\ \sigma_{tot,n}=\sigma_{rand,n}\;\mathrm{outside}\;\mathrm{the}\;\mathrm{vessel} \end{array}\right.$$

where *σ_PWD_* is the standard deviation related to the dispersion of the Doppler spectrogram, *σ_rand_* is the STD of a random distribution representative for the electronic noise that overlaps with the diagnostic representation in the spectral region, while *σ_th_* denotes the STD because of the parabolic profile assumption. As thoroughly described in [44,45], *σ_th_* depends in turn on the uncertainty contribution associated with the SV positioning relative to the vessel section, and the minimum SVL increment Δ*l*.

According to Equation (1), the discrepancy index is expected to be 0; otherwise, it can be inferred that the spectrogram images are affected by undesired SVL variation from the set value, and/or low registration accuracy due to the discrepancy between the actual position of the SV and that displayed on the B-mode image.

The main specifications adopted for the measurement method are listed in Table 3.

#### 2.2.2. Average Maximum Velocity Sensitivity

In the clinical setting, the maximum velocity measurement provides useful information concerning the hemodynamics of the heart and cardiovascular pathologies, e.g., heart valvular defects and arterial stenosis [18,54,55]. As a consequence, the accuracy of this measurement is one of the most investigated quality tests [18,32], recommended by different professional bodies such as the American Institute of Ultrasound in Medicine (AIUM) [35] and the Institute of Physics and Engineering in Medicine (IPEM) [34]. However, the measurement of the maximum Doppler scatterer velocity is affected by errors related to the UDS settings, including the insonation angle [56,57]. In this context, the Average Maximum Velocity Sensitivity (AMVS) was proposed in [46] as a novel sensitivity parameter to quantify the UDS response to flow velocity variations supplied by a Doppler reference device.

**Figure 3:**
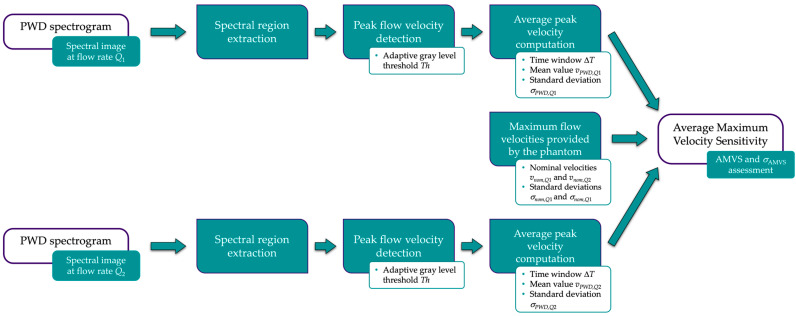
Block diagram of the measurement method for the average maximum velocity sensitivity assessment.

The implemented measurement method estimates AMVS by post-processing two PWD spectrogram images, each collected at a different constant flow rate (*Q*_1_ and *Q*_2_) while keeping the sample volume positioned on a sloped vessel flow axis of the reference Doppler device. The main processing steps of the image analysis-based method are shown in Figure 3 and summarized in the following.

The two spectral regions are extracted, and the adaptive threshold *Th* is applied as described above to derive the mono-dimensional signals of the maximum flow velocity through time from the images. For both signals, the average maximum velocity (*v_PWD_*_,*Q*1_ and *v_PWD_*_,*Q*2_ at *Q*_1_ and *Q*_2_, respectively), and the standard deviation (*σ_PWD_*_,*Q*1_ and *σ_PWD_*_,*Q*2_) are computed over a fixed time window Δ*T* corresponding to *L* spectral lines. At this point, AMVS is derived as follows:

(3) $$AMVS=\frac{∆v_{PWD}}{∆v_{nom}}=\frac{v_{PWD,Q2}-v_{PWD,Q1}}{v_{nom,Q2}-v_{nom,Q1}}$$

where Δ*v_PWD_* is the difference between the two measured average maximum velocities, whereas Δ*v_nom_* is the difference between the nominal maximum velocities (*v_nom_*_,*Q*1_ and *v_nom_*_,*Q*2_) given in the user’s guide of the reference device. Finally, the standard deviation of the parameter *σ*_AMVS_ is estimated by applying the uncertainty propagation law:

(4) $$\sigma_{AMVS}=AMVS\sqrt{{\left(\frac{\sigma_{∆vPWD}}{{∆v}_{PWD}}\right)}^{2}+{\left(\frac{\sigma_{∆vnom}}{{∆v}_{nom}}\right)}^{2}}$$

where *σ*_Δ*vPWD*_ is the standard deviation obtained by combining *σ_PWD_*_,*Q*1_ and *σ_PWD_*_,*Q*2_, while *σ*_Δ*vnom*_ is estimated by combining the flow velocity STD values (*σ_nom_*_,*Q*1_ and *σ_nom_*_,*Q*2_) derived from the phantom user’s guide.

According to Equation (3), the maximum sensitivity of the Doppler system is achieved when AMVS is equal to 1.

The main specifications adopted for the measurement method are listed in Table 4.

#### 2.2.3. Velocity Measurements Accuracy

Accuracy in blood velocity measurements is an essential requirement for assessing the health of the cardiovascular system [18]. Therefore, an objective measurement method for the Velocity Measurements Accuracy (VeMeA) assessment was developed to quantify the Doppler system accuracy in the estimation of the mean scatterer velocity. The definition of the VeMeA test was derived from the mean velocity estimation performance test recommended by the IPEM in [34], and its first investigation was performed in [38] for Color Doppler QA.

**Figure 4:**
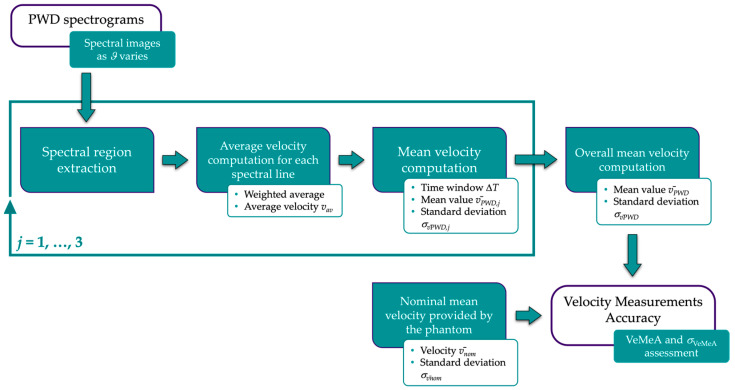
Block diagram of the measurement method for the velocity measurements accuracy assessment.

The implemented measurement method, preliminarily proposed in [40] for PWD testing, automatically estimates VeMeA by post-processing PWD spectrogram images. Data are required to be collected at three correction angle settings, i.e., insonation angle *ϑ* ± Δ*ϑ*, where Δ*ϑ* is the minimum correction angle variation, while keeping the sample volume positioned on the sloped vessel flow axis of the reference Doppler device. The main processing steps of the image analysis-based method are shown in Figure 4 and summarized in the following.

After the spectral region is identified and extracted from the acquired image, the average velocity *v_av_* is assessed on each spectral line of the diagnostic representation as expressed in the following weighted average [58]:

(5) $$v_{av}=\frac{\sum_{i=1}^{H}v_{i}{gl}_{i}}{\sum_{i=1}^{H}{gl}_{i}}$$

where *v_i_* is the *i*-th velocity value given by the *i*-th pixel starting from the baseline of the spectrogram, and *gl_i_* is the corresponding gray level. The corresponding mono-dimensional velocity trend through time is then retrieved based on the pre-set full-scale velocity. The mean velocity ${\bar{v}}_{PWD,j}$ (*j* = 1, …, 3) and the standard deviation $\sigma_{\bar{v}PWD,j}$ are computed over a fixed time window Δ*T* corresponding to *L* spectral lines. These processing steps are repeated for the three correction angles to compute the overall mean velocity ${\bar{v}}_{PWD}$ and estimate the combined standard deviation $\sigma_{\bar{v}PWD}$. At this point, VeMeA is derived as follows:

(6) $$VeMeA=\frac{\left|{\bar{v}}_{PWD}-{\bar{v}}_{nom}\right|}{{\bar{v}}_{nom}}$$

where ${\bar{v}}_{nom}$ is the nominal mean flow velocity as provided by the reference device. Finally, the uncertainty propagation law is applied to estimate the standard deviation of the test:

(7) $$\sigma_{VeMeA}\approx VeMeA\sqrt{{\left(\frac{\sigma_{\bar{v}PWD}}{{\bar{v}}_{PWD}}\right)}^{2}+{\left(\frac{\sigma_{\bar{v}nom}}{{\bar{v}}_{nom}}\right)}^{2}}$$

where $\sigma_{\bar{v}nom}$ denotes the flow velocity STD derived from the user’s guide of the phantom.

According to Equation (6), the maximum accuracy of the Doppler system is achieved when VeMeA is equal to 0.

The main specifications adopted for the measurement method are listed in Table 5.

#### 2.2.4. Lowest Detectable Signal

The measurement of the minimum detectable noise-free signal level is deemed a key factor in all Doppler measurements [35], as it is identified in the scientific literature as the sensitivity of the Doppler system [18,21]. In the clinical setting, maximum sensitivity refers to the depth from the ultrasound probe at which the UDS can detect Doppler signals from small vessels. In ref. [59], it is defined as the weakest Doppler shift signal the UDS can detect and display on the spectrogram, while from a metrological point of view, it can be identified as a detection limit rather than sensitivity [60]. In this context, the Lowest Detectable Signal (LDS) was proposed in [47,48] as a QA parameter to objectively quantify the flow detectability, expressed in dB.

**Figure 5:**
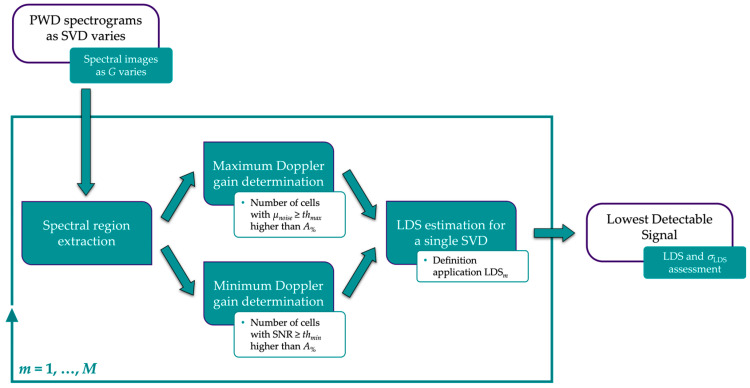
Block diagram of the measurement method for the lowest detectable signal assessment.

The measurement method automatically estimates the LDS by post-processing PWD spectrograms. The latter are required to be collected by adjusting the Doppler gain from minimum to maximum in steps of Δ*G*, keeping the length and depth of the sample volume fixed. The acquisition protocol needs to be repeated for *M* different depths by placing the sample volume on the sloped vessel flow axis of the reference Doppler device. The mathematical formulation derived for the LDS, considering the main factors influencing it, is applied as follows:

(8) $$LDS=\frac{1}{M}\sum_{m=1}^{M}{LDS}_{m}=\frac{1}{M}\sum_{m=1}^{M}\left(2\alpha f_{D}z_{m}+G_{max,m}-G_{min,m}\right)$$

where *α* (dB·cm^−1^·MHz^−1^) is the nominal attenuation coefficient of the medium, *f_D_* (MHz) is the nominal Doppler frequency of the probe, *z* (cm) is the depth of the sample volume, *G_max_* (dB) is the maximum Doppler gain before the occurrence of non-negligible noise, and *G_min_* (dB) is the minimum Doppler gain at which the intensity of the spectrogram is close to zero, i.e., lack of signal. The main processing steps of the image analysis-based method are shown in Figure 5 and summarized in the following.

Each set of PWD spectrograms for a fixed SVD undergoes spectral region identification and extraction before *G_max_* and *G_min_* in Equation (8) are determined.

**Figure 6:**
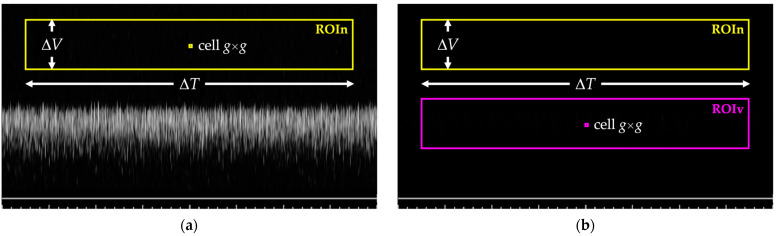
Example of ROIn and ROIv positioning on the spectral images to determine the maximum Doppler gain *G_max_* (**a**) and the minimum Doppler gain *G_min_* (**b**).

- For the first gain value, a region of interest, referred to as ROIn, is drawn at the top of each spectral image where the noise is expected to appear (Figure 6a). The size in pixels of the region is derived by considering a fixed window on the time axis and one on the velocity axis, Δ*T* and Δ*V*, respectively. ROIn is divided into cells of *g* × *g* pixels, and the average gray level *μ_noise_*_,*p*_ is computed for each cell. Therefore, a further region of interest (ROIn2) is obtained, one for each spectral image, i.e., one for each Doppler gain setting. At this point, *G_max_* is determined as the lowest gain for which the number of cells in ROIn2 with *μ_noise_*_,*p*_ ≥ *th_max_* is greater than *A*_%_ (expressed as a percentage of the total number of cells).
- For the second gain value, a further region of interest, referred to as ROIv, is drawn on the spectral images in addition to ROIn. As shown in Figure 6b, it maintains the same size (Δ*T* × Δ*V*) but is positioned to include pixels that on the velocity scale correspond to the nominal maximum velocity set on the reference device. The average gray level *μ_NOISE_* of ROIn is computed as the noise level, whereas ROIv is divided into cells of *g* × *g* pixels, and the average gray level *μ_signal_*_,*p*_ is computed for each cell, as in the previous case. Then, a Signal-to-Noise Ratio (SNR) matrix of elements *g* × *g* is derived, one for each spectral image, i.e., one for each Doppler gain setting:

(9) $${SNR}_{p}=\frac{\mu_{signal,p}}{\mu_{NOISE}}$$

At this point, *G_min_* is determined as the lowest gain for which the number of cells with SNR*_p_* ≥ *th_min_* is greater than *A*_%_.

It is worth noting that a specific unit conversion procedure is required if the diagnostic system under consideration does not provide Doppler gain in dB [47].

Therefore, the lowest detectable signal LDS*_m_* is assessed by applying Equation (8) for the *m*-th depth of the sample volume, while both the attenuation coefficient of the medium and the Doppler frequency of the probe are fixed. Finally, the mean value is computed to retrieve the overall LDS parameter, and the standard deviation *σ*_LDS_ is estimated.

The main specifications adopted for the measurement method are listed in Table 6.

### 2.3. Data Acquisition Protocol

All linear array transducers were held in place on the scanning surface of the reference device by a probe holder [50] during the whole acquisition phase. In addition, a coupling gel was always used to maximize ultrasound energy transmission.

As shown in Table 7, the flow rate provided by the Doppler phantom was adjusted according to the parameter to be assessed while keeping the flow mode constant. As regards the LDS, the lowest and most stable flow rate was set [47,48], while flow rates in the medium regime were adopted for the others. Based on preliminary results obtained in [46], a flow rate step of 1.5 mL·s^−1^ was chosen for the AMVS assessment.

PWD spectrograms were acquired on the diagonal segment of the phantom vessel by varying the length and depth of the sample volume as well as the correction angle according to the parameter. Table 8 summarizes the sample volume settings for each parameter, depending on the ultrasound diagnostic system.

Finally, as regards data acquisition for LDS assessment, it should be noted that different Doppler gain steps Δ*G* were considered depending on the ultrasound system, as shown in Table 9.

### 2.4. Parameter Scaling

A scaling procedure was required because the expected value differs per parameter (Table 10). As in [38,39,40], parameter-specific mapping equations were applied to express them in a standard range from 0 to 1, where 1 represents the optimal value. Scaled values, denoted by the symbol (*), for the VPDI, AMVS, VeMeA and LDS tests were computed as follows:

(10) VPDI*=e−VPDIκ

(11) $${AMVS}^{*}=1-\left(\left|AMVS-1\right|\right)$$

(12) $${VeMeA}^{*}=\frac{1}{1+VeMeA}$$

(13) $${LDS}^{*}=\frac{LDS}{B_{max}}=\frac{LDS}{2\alpha f_{D}z_{max}}$$

The exponential function for VPDI scaling was adopted to emphasize small differences between outcomes closer to the expected value. The constant *κ* in Equation (10) was estimated based on the first results obtained in [44,45] assuming a 75% reduction in the expected value when the VPDI is equal to 3, i.e., *κ* = 2.2. On the other hand, LDS scaling was carried out assuming *B_max_* in Equation (13) as the maximum expected attenuation, expressed as a function of the (mean) attenuation of the medium *α*, the operating Doppler frequency *f_D_* of the probe, and the maximum depth *z_max_* of the phantom vessel from the scanning surface. Therefore, LDS scaling depends on the characteristics of the reference device used.

## 3. Measurement Uncertainty Analysis

The uncertainty analysis allows for assessing the measurement quality and the compatibility among measurements. Therefore, custom-written algorithms were implemented using MATLAB routines to estimate the uncertainty contribution associated with each image analysis-based method implemented, as in [38,39,40]. The estimation was carried out through the Monte Carlo Simulation (MCS), which can be viewed as a statistical method for propagating distributions by performing random sampling from probability density functions (PDFs) [61,62,63,64]. A simulation was run through 10^5^ iterations for each combination of UDS, pre-set, and parameter. The main quantities upon which the four parameters depend on were identified to assign them an input distribution (expressed as mean ± STD) as listed in Table 11:

1. For both the VPDI and AMVS, simulations were performed by assuming a distribution of the adaptive threshold *Th_g_*, and the *L* spectral lines (corresponding to the time window Δ*T*) were randomized, at each iteration without repetition, among all those constituting the spectral image. The standard deviation of each output distribution *σ_MCS_* was estimated and, for AMVS alone, this was combined with the repeatability STD *σ*_AMVS_ retrieved in Section 2.2.2;
2. For VeMeA, only spectral line randomization was applied, and *σ_MCS_* was combined with the repeatability standard deviation *σ*_VeMeA_ in Section 2.2.3;
3. For LDS, an MCS was run for each SVD setting by assigning a distribution to the quantities in Equation (8), i.e., the attenuation coefficient of the phantom TMM, the sample volume depth, and the maximum as well as the minimum Doppler gain. The standard deviation *σ_α_* was retrieved from the user’s guide of the reference device assuming a 95% confidence level, *σ_z_* was derived from the sample volume depth resolution of 1 mm, while *σ_Gmax_* and*σ_Gmin_* were estimated from the Doppler gain step Δ*G* taken at the acquisition phase. Given the narrow bandwidth of the transmitted pulse, the uncertainty related to the Doppler frequency was considered negligible, and therefore no probability density function was assigned to *f_D_*. Finally, the STD of the parameter *σ*_LDS_ was estimated as the mean of the standard deviations of the *M* output distributions.

## 4. Results

Experimental results of the test parameters retrieved for each ultrasound system in both working conditions (pre-set A and B) are shown in Table 12. For the comparison of the results, a compatibility analysis was performed according to the criterion in [65].

Focusing on the VPDI results, UDS2 showed the lowest values, i.e., closer to the expected ones, independently of the pre-set (0.15 ± 0.09 and 0.08 ± 0.01). On the other hand, a discrepancy in the results was found between the two pre-sets of the other ultrasound systems: higher VPDI values were observed for pre-set B of UDS1 and UDS3, and for pre-set A of UDS4 and UDS5. The result deviating most from the expected value was found for UDS5 in pre-set A (2.67 ± 0.17).

AMVS outcomes were all close to the expected value (maximum sensitivity is reached when AMVS is equal to 1), and general compatibility among ultrasound systems was observed, independently of the working condition.

Conversely, VeMeA results deviated significantly from the expected value, as the parameter was expected to be as close to 0, i.e., maximum accuracy. Compatibility was not always guaranteed by comparing diagnostic systems with each other, although it was maintained between pre-set A and B of the same UDS.

As regards LDS outcomes, it was not possible to make a direct comparison with the corresponding expected value, because the latter, as mentioned above, is a conventional value that depends on the characteristics of the reference device. General compatibility was noticed between UDSs, while compatible results were always found by comparing the two pre-sets of the same ultrasound system, except for UDS4. For pre-set B of the latter system, LDS could not be assessed because the minimum Doppler gain in Equation (8) was not determined. This was due to the presence of a weak signal detected by the measurement method even at the minimum Doppler gain setting. In addition, since noise appeared at gain settings close to the minimum, this issue could not be overcome by acquiring data at greater sample volume depths.

Scaled experimental outcomes of the four parameters were derived according to the scaling procedure described in Section 2.4 to combine and represent them on the Kiviat diagram. They are listed in Table 13 for each ultrasound system in both working conditions (pre-set A and B), together with the corresponding diagram area (expressed as mean ± STD). The latter was normalized to the gold standard area, i.e., the total area of the polygon resulting when all parameters assume the optimal value. By applying this further step, the diagram areas were also expressed in a standard range from 0 to 1, where 1 represents the optimal value.

Keeping the considerations above on unscaled outcomes, scaled outcomes allow a proper and effective comparison between test parameters. Among all, AMVS* results were the closest to 1, but with the highest standard uncertainties, independently of the ultrasound system and pre-set. As mentioned above, a discrepancy in the VPDI* results was generally found between the two pre-sets. On the other hand, the parameters that significantly deviated from the optimal value were VeMeA* and LDS*. It is worth noting that lower LDS* values were obtained for UDS5 after scaling compared with the other ultrasound systems. This behavior, accounted for by the adopted scaling (Equation (13)), was expected due to the greater attenuation affecting high-frequency ultrasound waves. As shown in Table 1, the probe equipped on UDS5 was the only one operating at a slightly higher Doppler frequency.

Focusing on the diagram areas (Table 13), general compatibility was observed between the ultrasound systems, and compatible performance between the two working conditions was always found for all UDSs except UDS4. It should be pointed out that the normalized area of UDS4 in pre-set B (0.50 ± 0.10) was computed by assuming the same scaled result for LDS in pre-set A (0.46 ± 0.02). This choice was made based on the compatibility of the results observed between the two working conditions of the same UDS, as shown in Table 12 and Table 13.

**Figure 7:**
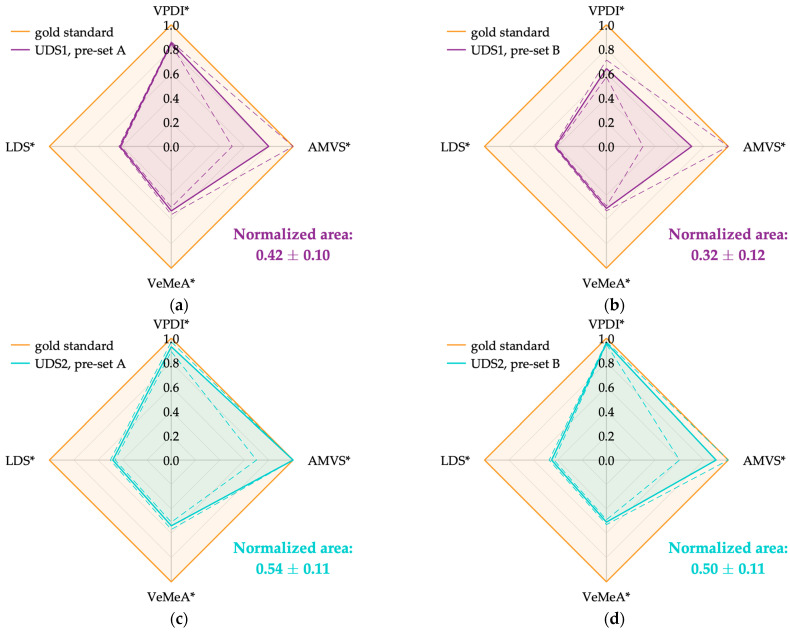
Kiviat diagrams for UDS1 (**a**,**b**), UD2 (**c**,**d**) according to the working condition: pre-set A (**a**,**c**) and pre-set B (**b**,**d**).

**Figure 8:**
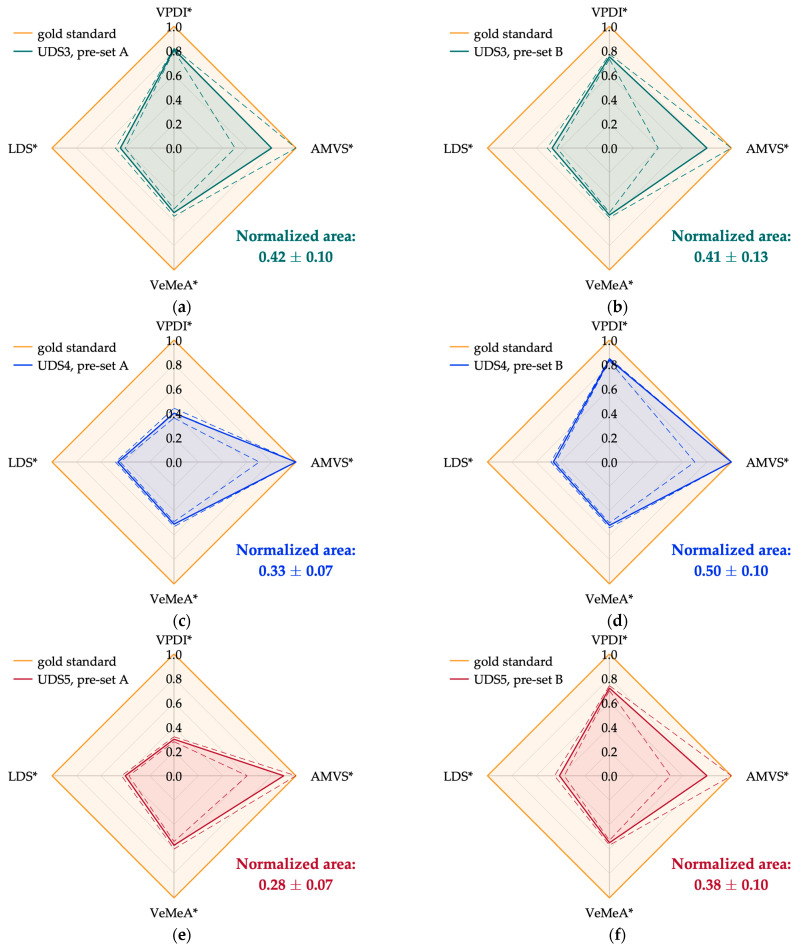
Kiviat diagrams for UDS3 (**a**,**b**), UDS4 (**c**,**d**), and UD5 (**e**,**f**) according to the working condition: pre-set A (**a**,**c**,**e**) and pre-set B (**b**,**d**,**f**).

Finally, Kiviat diagrams representative of the five ultrasound systems in both pre-sets are shown in Figure 7 and Figure 8.

## 5. Discussion and Conclusions

The present study aims to investigate the Kiviat diagram-based integrated approach, recently proposed to effectively combine the contribution of experimental parameters and quantify the overall UDS performance, for the comparison of PWD diagnostics systems. Four test parameters were objectively assessed by custom-written measurement methods based on image analysis: the velocity profile discrepancy index, average maximum velocity sensitivity, velocity measurements accuracy, and lowest detectable signal. Parameter-specific mapping equations were applied to express the parameters above in a standard range and represent them on the Kiviat plot. Five linear array probes equipped on as many ultrasound diagnostic systems intended for use in clinical care were tested in two working conditions using a reference device for Doppler applications.

Based on the experimental outcomes, it is worth noting that no significant discrepancies were found between the two working conditions for AMVS, VeMeA, and LDS parameters (Table 12 and Table 13). Therefore, these parameters can be considered independent of the ultrasound system pre-set. Conversely, well-defined behavior between pre-set A and B for the VPDI cannot be inferred, so further investigations should also be performed on the parameter to determine each contribution of the two sources of error (sample volume length and registration accuracy).

The Kiviat plot (Figure 7 and Figure 8) turned out to be a useful tool for combining the experimental parameters while preserving the relationship between them and estimating a single index, i.e., the diagram area, which provided an immediate assessment of the overall performance of PWD systems. In this regard, the five ultrasound systems included in this study showed comparable performance with each other (Table 13). This was expected since they were brand-new systems of the same technological level. However, it was still possible to appreciate slight discrepancies among some of them, e.g., UDS5 and UDS2, for which the lowest (0.28) and highest (0.54) mean areas were found, respectively. The promising outcomes of this comparative study suggest that the proposed approach could be useful in clinical and industrial settings for scheduled QA of Doppler equipment and for comparing UDSs from different vendors or technological levels. In the near future, it would be interesting to investigate how much image quality degradation affects the shape of the diagram. Determining the sensitivity and specificity of this integrated approach could help us to understand whether it can transduce a change in one or more parameters in significant discrepancies between areas of the diagram. As a last remark, further studies should be carried out by increasing the number of experimental parameters and UDSs, also including different probe models and phantoms (e.g., [66,67]). In the future, further studies are going to be carried out to confirm the effectiveness and limitations of some test parameters, also considering new ultrasound technologies and strategies applied to diagnostics [68,69], as well as to provide a combination of the outcomes from different modalities (e.g., B-mode, pulsed wave Doppler, Color Doppler) to determine a whole quality index.

## Figures and Tables

**Table 1:** General PWD settings of the ultrasound systems per working condition.

Setting	Pre-Set	UDS1	UDS2	UDS3	UDS4	UDS5
Field of view (cm)	A and B	7	7	7	7	7
Doppler frequency (MHz)	A and B	5.0	5.0	5.0	5.0	5.5
Wall filter	A	medium	high	medium	low	low
	B	minimum	minimum	minimum	minimum	minimum
Compression	A	medium	low	medium	medium	low
	B	minimum	minimum	minimum	minimum	minimum
Power	A	maximum	maximum	high	high	high
	B	maximum	maximum	maximum	maximum	maximum
Doppler baseline	A and B	minimum	minimum	minimum	minimum	minimum
Spectrogram resolution (px × px)	A and B	960 × 1280	478 × 900	455 × 1210	345 × 1690	480 × 1365
Spectrogram duration (s)	A and B	9.5	8	12	~12	~11

**Table 2:** Technical specifications of the reference device.

Feature	Specification
Phantom model	Doppler 403^TM^ flow phantom (Sun Nuclear corporation, Middleton, WI, USA)
Horizontal vessel	5.0 ± 0.2 mm inner diameter at 2 cm depth
Diagonal vessel	5.0 ± 0.2 mm inner diameter at 40° from 2 to 16 cm deep
Attenuation coefficient	0.70 ± 0.05 dB·cm^−1^·MHz^−1^
Scanning surface	patented composite film
TMM	patented High Equivalency Gel^TM^
TMM sound speed	1540 ± 10 m·s^−1^
BMF sound speed	1550 ± 10 m·s^−1^
Flow range	(1.7–12.5) ± 0.4 mL·s^−1^
Flow rate	customizable, constant, and pulsatile
Dimensions (case)	28.0 × 30.5 × 22.0 cm

**Table 3:** Main measurement method specifications assumed for VPDI assessment.

Feature	Symbol	Value
Adaptive threshold	*Th*	10% of the maximum gray level displayed
Time window	Δ*T*	7 s
Number of spectral lines	*L*	varies with the resolution and duration of the PWD spectrogram
Minimum SVL increment	Δ*l*	1 mm
Number of SV depths	*N*	7

**Table 4:** Main measurement method specifications assumed for AMVS assessment.

Feature	Symbol	Value
Adaptive threshold	*Th*	10% of the maximum gray level displayed
Time window	Δ*T*	7 s
Number of spectral lines	*L*	varies with the resolution and duration of the PWD spectrogram

**Table 5:** Main measurement method specifications assumed for VeMeA assessment.

Feature	Symbol	Value
Time window	Δ*T*	7 s
Number of spectral lines	*L*	varies with the resolution and duration of the PWD spectrogram

**Table 6:** Main measurement method specifications assumed for LDS assessment.

Feature	Symbol	Value
Time window	Δ*T*	7 s
Velocity window	Δ*V*	15 cm·s^−1^
Cell size	*g* × *g*	6 × 6 px
Threshold for *G_max_* determination	*th_max_*	3
Threshold for *G_min_* determination	*th_min_*	2
Percentage of the total number of cells	*A*_%_	1%
Number of SV depths	*M*	5

**Table 7:** Flow rate set per parameter and corresponding nominal average and maximum velocities.

Parameter	Flow Rate (mL·s^−1^)	Average Velocity (cm·s^−1^)	Maximum Velocity (cm·s^−1^)
VPDI	7.0	35.7	71.3
AMVS	5.5 and 7.0	28.0 and 35.7	56.0 and 71.3
VeMeA	7.0	35.7	71.3
LDS	2.0	10.2	20.4

**Table 8:** Sample volume settings of the ultrasound systems per QA parameter.

Setting	Parameter	UDS1	UDS2	UDS3	UDS4	UDS5
Sample volume length (mm)	VPDI	1.0	1.0	1.0	1.0	1.0
	AMVS	1.0	1.0	1.0	1.0	1.0
	VeMeA	1.0	1.0	1.0	1.0	1.0
	LDS	1.5	2.0	1.5	1.5	1.5
Sample volume depth (mm)	VPDI	35 to 41; 1 mm spaced	35 to 41; 1 mm spaced	35 to 41; 1 mm spaced	35 to 41; 1 mm spaced	35 to 41; 1 mm spaced
	AMVS	40	40	40	40	40
	VeMeA	40	40	40	40	40
	LDS	42 to 50; 2 mm spaced	38 to 46; 2 mm spaced	48 to 56; 2 mm spaced	62 to 70; 2 mm spaced	42 to 50; 2 mm spaced
Correction angle (°)	VPDI	50	50	50	50	50
	AMVS	50	50	50	50	50
	VeMeA	50 ± 1	50 ± 1	50 ± 1	50 ± 2	50 ± 1
	LDS	50	50	50	50	50

**Table 9:** Doppler gain settings per ultrasound system in LDS assessment.

Ultrasound System	Doppler Gain		
	Range	Gain Step Δ*G*	Measurement Unit
UDS1	from 0 to 50	2	dB
UDS2	from 0 to 85	5	dB
UDS3	from 0 to 100	2	a.u. ^(1)^
UDS4	from 0 to 100	4	%
UDS5	from 0 to 100	2	a.u. ^(1)^

^(1)^ a.u. = arbitrary units.

**Table 10:** Summary of the expected values for each QA parameter.

Test Parameter	Acronym	Expected Value
Velocity profile discrepancy index	VPDI	0
Average maximum velocity sensitivity	AMVS	1
Velocity measurements accuracy	VeMeA	0
Lowest detectable signal	LDS	*B_max_* ^(1)^

^(1)^ *B_max_* is a conventional value that depends on the phantom used.

**Table 11:** Probability density functions assigned in MCSs for estimating the measurement uncertainty of implemented methods by parameter.

VPDI and AMVS	Symbol	PDF	Mean ± STD
Adaptive gray level threshold	*Th_g_* ± *σ_Thg_*	uniform	(10% ± 1%) of *gl_max_* ^(1)^
Spectral line randomization	-	-	-
**VeMeA**			
Spectral line randomization	-	-	-
**LDS**			
TMM attenuation (dB·cm^−1^·MHz^−1^)	*α* ± *σ_α_*	normal	0.700 ± 0.025
Sample volume depth (mm)	*z* ± *σ_z_*	uniform	*z* ± 0.3
Maximum Doppler gain	*G_max_* ± *σ_Gmax_*	uniform	*G_max_* ± Δ*G*/2√3
Minimum Doppler gain	*G_min_* ± *σ_Gmin_*	uniform	*G_min_* ± Δ*G*/2√3

^(1)^ *gl_max_* = maximum gray level displayed.

**Table 12:** Test parameters outcomes (mean ± STD) according to the ultrasound diagnostic system and working condition.

Ultrasound System	Pre-Set	VPDI	AMVS	VeMeA	LDS (dB)
UDS1	A	0.35 ± 0.03	1.2 ± 0.3	0.90 ± 0.09	47.4 ± 1.4
	B	1.00 ± 0.24	1.3 ± 0.4	0.96 ± 0.09	47.0 ± 1.4
UDS2	A	0.15 ± 0.09	1.0 ± 0.3	0.87 ± 0.09	53.4 ± 2.3
	B	0.08 ± 0.01	0.9 ± 0.3	0.96 ± 0.09	50.4 ± 2.3
UDS3	A	0.47 ± 0.04	1.2 ± 0.3	0.88 ± 0.09	49 ± 5
	B	0.64 ± 0.05	1.2 ± 0.4	0.82 ± 0.08	53 ± 5
UDS4	A	2.01 ± 0.23	1.0 ± 0.3	0.95 ± 0.09	51.7 ± 2.3
	B	0.38 ± 0.02	1.0 ± 0.3	0.94 ± 0.09	N.A. ^(1)^
UDS5	A	2.67 ± 0.17	1.1 ± 0.3	0.75 ± 0.08	49 ± 4
	B	0.72 ± 0.07	1.2 ± 0.3	0.81 ± 0.08	50 ± 5

^(1)^ N.A. = not available.

**Table 13:** Scaled test parameters outcomes (mean ± STD) and normalized diagram areas (mean ± STD) according to the ultrasound diagnostic system and working condition.

Ultrasound System	Pre-Set	VPDI*	AMVS*	VeMeA*	LDS*	Normalized Area
UDS1	A	0.85 ± 0.01	0.8 ± 0.3	0.53 ± 0.03	0.42 ± 0.01	0.42 ± 0.10
	B	0.64 ± 0.07	0.7 ± 0.4	0.51 ± 0.02	0.42 ± 0.01	0.32 ± 0.12
UDS2	A	0.93 ± 0.04	1.0 ± 0.3	0.54 ± 0.03	0.48 ± 0.02	0.54 ± 0.11
	B	0.96 ± 0.01	0.9 ± 0.3	0.51 ± 0.02	0.45 ± 0.02	0.50 ± 0.11
UDS3	A	0.81 ± 0.01	0.8 ± 0.3	0.53 ± 0.03	0.44 ± 0.04	0.42 ± 0.10
	B	0.75 ± 0.02	0.8 ± 0.4	0.55 ± 0.02	0.47 ± 0.04	0.41 ± 0.13
UDS4	A	0.40 ± 0.04	1.0 ± 0.3	0.51 ± 0.02	0.46 ± 0.02	0.33 ± 0.07
	B	0.84 ± 0.01	1.0 ± 0.3	0.52 ± 0.02	N.A. ^(1)^	0.50 ± 0.10 ^(2)^
UDS5	A	0.30 ± 0.02	0.9 ± 0.3	0.57 ± 0.03	0.40 ± 0.03	0.28 ± 0.07
	B	0.72 ± 0.02	0.8 ± 0.3	0.55 ± 0.02	0.41 ± 0.04	0.38 ± 0.10

^(1)^ N.A. = not available; ^(2)^ The area was computed on the assumption that LDS* in pre-set B was equal to that assessed in pre-set A of the same UDS.

## Data Availability

Data are contained within the article.
